# Antihypertensive Effects of Virgin Olive Oil (Unfiltered) Low Molecular Weight Peptides with ACE Inhibitory Activity in Spontaneously Hypertensive Rats

**DOI:** 10.3390/nu12010271

**Published:** 2020-01-20

**Authors:** Juan María Alcaide-Hidalgo, Miguel Romero, Juan Duarte, Eduardo López-Huertas

**Affiliations:** 1 Group of Antioxidants and Free Radicals in Biotechnology, Food and Agriculture, Estación Experimental Zaidín, Consejo Superior de Investigaciones Científicas (CSIC), Profesor Albareda 1, 18008 Granada, Spain; juanmaria.alcaide@eez.csic.es; 2 Pharmacology Department, Faculty of Pharmacy, University of Granada, CIBER-Enfermedades Cardiovasculares (CiberCV), 18071 Granada, Spain; miguelr@ugr.es (M.R.); jmduarte@ugr.es (J.D.)

**Keywords:** unfiltered virgin olive oil, peptides, ACE, hypertension, spontaneously hypertensive rats

## Abstract

The low molecular weight peptide composition of virgin olive oil (VOO) is mostly unknown. We hypothesised that unfiltered VOO could possess low molecular weight peptides with antihypertensive activity. We produced unfiltered VOO and obtained a water-soluble peptide extract from it. The peptides were separated by size-exclusion using fast protein liquid chromatography, and the low molecular weight fraction was analysed by nanoscale liquid chromatography-Orbitrap coupled with tandem mass spectrometry and de novo sequencing. We selected 23 peptide sequences containing between 6 and 9 amino acids and molecular masses ranging 698–1017 Da. Those peptides were chemically synthesised and their angiotensin-converting enzyme (ACE) inhibitory activity was studied in vitro. Seven peptides showed a strong activity, with half maximal inhibitory concentration (IC50) <10 µm. The antihypertensive effects of the four most active synthesised ACE inhibitor peptides were studied in spontaneously hypertensive rats (SHR). Acute oral administration of synthetic peptides RDGGYCC and CCGNAVPQ showed antihypertensive activity in SHR. We conclude that unfiltered VOO naturally contains low molecular weight peptides with specific ACE inhibitory activity and antihypertensive effects in SHR.

## 1. Introduction

Virgin olive oil (VOO) is the main source of fat of the Mediterranean diet [1]. VOO is composed mainly of two fractions: the fat fraction, which predominantly contains fatty acids (about 80% oleic acid) in the form of triglycerides, is 97–99% of the olive oil composition; and the unsaponifiable fraction, also named “minor components,” which represent 1–3% of the composition. This fraction is a combination of nutrients which are transferred from the olives to the oil during the extraction of olive oil and mainly contains polyphenols, tocopherols, sterols, terpenic acids, chlorophyll, and carotenoids [2,3]. The minor components content of VOO is determined by a large number of factors such as the variety of olive tree, the fruit ripening, irrigation, extraction technology, storage, etc. Virgin olive oil (VOO) has been demonstrated to be beneficial for the cardiovascular system by reducing blood cholesterol levels because of its oleic acid content [4] and also by protecting blood lipids from oxidations because of the antioxidant action of its specific polyphenols [5]. In addition, a few human studies have also shown that VOO may produce beneficial effects in the control of blood pressure in normal and hypertensive subjects [6,7,8,9]. These antihypertensive effects of VOO have also been associated with the high oleic acid content [10,11] and to its content in specific polyphenols [12,13,14,15,16,17]. Triterpenoid acids have shown positive effects in animal models of hypertension but so far have failed to show antihypertensive effects in humans [18]. One of the mechanisms that regulate blood pressure is the renin-angiotensin system, in which the angiotensin-converting enzyme (ACE) plays an important part. Inhibition of the ACE is a widely used strategy for the treatment of hypertension [19] so the food and pharmaceutical industries are always interested in new sources of ACE inhibitors with antihypertensive activity [20]. The great majority of ACE inhibitory peptides reported so far in the scientific literature, are obtained from dietary proteins of different origins by the action of several types of proteases (reviewed in [21]). However, bioactive peptides with defined functions can also be present in foods naturally [22].

In recent years, proteins and peptides have been reported to be part of the family of minor components of olive oil. The values of protein concentration described in olive oil differ with the use of different conditions and extraction methodologies and ranged between 0.1 and 43 mg/kg of oil [23,24,25,26,27]. Our previous work has shown that unfiltered VOO contains peptides of low molecular weight. We also reported that a water-soluble extract obtained from this unfiltered VOO possesses ACE inhibitory activity and antihypertensive effect in an animal model of hypertension (spontaneously hypertensive rats, SHR) [28]. In the present study we report the presence of low molecular weight peptides with ACE inhibitory activity in unfiltered VOO and we study the antihypertensive effects of specific peptides in SHR. We conclude that, unfiltered VOO naturally contains low molecular weight peptide sequences with specific ACE inhibitory and antihypertensive activity with potential nutritional and pharmaceutical applications.

## 2. Materials and Methods

### 2.1. Plant Material and Unfiltered Olive Oil Extraction

Olives (*Olea europea*) of the Picual variety were handpicked from healthy olive trees as described in [29]. Olive oil was extracted by using a two-phase production extraction plant as indicated in [28]. The resulting unfiltered virgin olive oil was stored at −20 °C.

### 2.2. Preparation of Olive Oil Extract Containing Peptides

Olive oil peptides were extracted from our olive oil samples as described in [28]. The water-soluble peptide fractions obtained were used for the experiments.

### 2.3. Fractionation of Peptides by Size-Exclusion Chromatography

**Figure 1:**
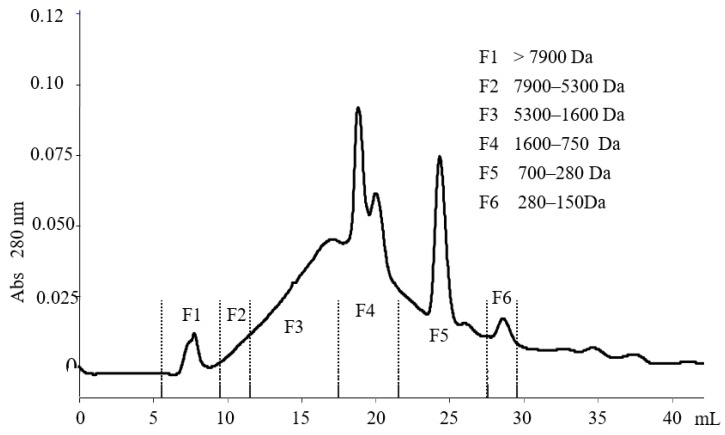
Separation of peptides extracted from unfiltered virgin olive oil by gel filtration chromatography and FPLC.

Gel filtration chromatography was used to separate the peptides extracted with the above method, using fast protein liquid chromatography (FPLC) with an “ÄKTApurifier” (GE Healthcare, UK) system equipped with a Superdex Peptide 10/300 GL (GE Healthcare, reference number 17-5176-01, length 30 cm, internal diameter 10 mm, average particle size 13 μm). The molecular size standards used to calibrate the gel filtration column and the conditions used to purify the samples were previously described in [28]. A volume of 200 µL of the samples and standards were injected into the ÄKTApurifier for analysis. After each injection, seventeen fractions of 2-mL each were collected. Using as a reference the chromatographic profile recorded at 280 nm (see Figure 1), the fractions were pooled into six groups (F1–F6). A rotary evaporator was used to dry the fractions until further analysis.

### 2.4. Analysis of Peptide Fractions by Nano LC-Orbitrap-MS/MS and de Novo Sequencing

The sequence of water-soluble peptides extracted from the olive oil was determined by nanoscale liquid chromatography-Orbitrap coupled with tandem mass spectrometry (nanoLC-Orbitap-MS/MS) and de novo sequencing. For this, approximately 1 μg of total protein was injected in the system and analysed in triplicates. Before injection, the samples were diluted with 0.1% formic acid in Milli Q water to a final peptide concentration of 0.25 mg/mL. The peptides were separated onto a C-18 reversed phase nano-column (75 μm id × 15 cm; 3 μm, Nikkyo Technos Co., Tokyo, Japan) coupled with a nano-precolumn (100 μm id × 2 cm, 5 μm, Thermo Fisher Scientific, Waltham, MA, USA). The chromatographic separation was performed with 0.1% formic acid in Milli Q water (phase A) and 0.1% formic acid in acetonitrile (phase B), using the following gradient: 0 to 5% B in 4 min, 5 to 15% of B in 60 min, 15 to 35% B in 60 min and 35 to 95% B in 10 min, maintained for 20 min. A flow rate of 300 nL/min was used to elute peptides for real time ionisation and peptide fragmentation on an LTQ-Orbitrap Velos Pro mass spectrometer (Thermo Fisher). An enhanced Fourier transform MS/MS-resolution spectrum (resolution = 30,000 full width at half maximum) was obtained followed by a data dependent MS/MS scan. The data dependent MS/MS event consists of collision-induced dissociation fragmentation (35% normalised collision energy) and ion trap-MS/MS acquisition from the most intense ten parent ions with a charge state rejection of +1 (Z = 1) and dynamic exclusion of 0.5 min, which is typically used for peptide identification. Interferences with other possible organic compounds was reduced by selecting only precursor ions with a minimum charge of +2 (Z = 2) or higher.

PEAKS studio 7 software and de novo sequencing was used characterise the peptides [30]. The following parameters were used for the analysis: parent mass error tolerance and fragment mass error tolerance were set at 10.0 ppm and 0.8 Da, respectively; enzyme: none; variable modifications: oxidation of methionine. The following setting were used to filter the results: de novo total local confidence (TLC) ≥5 and de novo average local confidence (ALC) was initially set at ≥60%, as described in previous reports [31]. Peptide identification was accepted if they appeared in at least two independent samples as in [32].

### 2.5. Selection of Potentially Active ACE Inhibitor Peptides

We looked for potentially active ACE inhibitor peptides among the sequences detected in the analysis, as follows. First, the peptide sequences were considered only if they were independently detected in at least two different sample injections and also if their ALC was above 70%. We investigated the presence of ACE inhibitory peptide fragments in the sequences and quantified for their theoretical inhibitory activity, using the BIOPEP database (http://www.uwm.edu.pl/biochemia/index.php/pl/biopep), which contains referenced data from 3793 bioactive peptides (as of July 2019), including 924 inhibitors of ACE [33]. The database uses parameters A and B to calculate the potential biological activity of a peptide sequence. A, is the number of sequences with previously reported ACE inhibitory activity divided by the total number of amino acids (N) of the peptide sequence. B, is the potential biological activity of the peptide (µm^−1^) calculated using published data of activity of the ACE inhibitor sequence motif (s) present in the peptide [34]. The values of A and B were used to select peptides with potential ACE inhibitory activity. The final selection was made using structural requirements such as molecular mass (ACE inhibitory peptides have usually less than 12 amino acids) and the presence of hydrophobic or charged amino acids in the C-terminal tripeptide sequence, which have an influence in ACE inhibitory activity [20].

### 2.6. Peptide Synthesis

Peptides identified in olive oil with potential ACE inhibitory activity were synthesised by CASLO ApS (Scion Denmark Technical University, Lyngby, Denmark) and used for in vitro and in vivo experiments. MALDI-TOF was used to certify the purity of the peptides obtained by synthesis. (not shown).

### 2.7. Peptide Concentration Determination

The concentration of peptides was tested in the samples by fluorescence using the FluoroProfile quantification kit (Sigma-Aldrich, St. Louis, MO, USA).

### 2.8. Determination of Angiotensin-Converting Enzyme Inhibitory Activity

The ACE inhibitory activity of the samples was studied using the methodology specified in [35], with some modifications [28]. The ACE inhibitory activity is shown as IC_50_, or the concentration needed to inhibit the activity of the enzyme by 50%.

### 2.9. Antihypertensive Activity of Olive Oil Peptides in SHR

The antihypertensive effect of synthetic peptides No. 1 (RDGGYCC), No. 2 (LEEFCC), No. 3 (HCGCNTH) and No. 4 (CCGNAVPQ) was studied on the systolic blood pressure (SBP) and diastolic blood pressure (DBP) using the spontaneously hypertensive rat (SHR) animal model of hypertension. The tail-cuff method was used to measure SBP and DBP [36] as described in [28]. We selected a dose of synthetic peptides in the range described in previous studies of antihypertensive effects of peptides with ACE inhibitory activity [37,38]. SBP and DBP data was always collected by the same technician. A period of training of two weeks was set to allow the animals to be habituated to this methodology [39]. Forty-eight male SHR (age 19–21 weeks) were used. The average BW of the animals was 322.0 ± 6.0 g. The synthetic peptides were dissolved in water and were administered orally to the animals (about 1 mL per animal). The ACE inhibitor drug Captopril (Sigma, St. Louis, MO, USA) was used as a positive control and water was used as a negative control. The SHR were distributed randomly into 6 study groups (*n* = 8 per group) and received an oral dose of one of the four synthetic peptides (25 mg/kg of BW), or a dose of Captopril (50 mg/kg of BW) or water (1 mL). SBP and DBP were measured in the rats at baseline (time 0) and at 2, 4, 8 and 24 h post-administration of the peptides or controls. Measurements were considered valid when at least six consecutive SBP and DBP data were similar. The Bioethical Committee of Granada University (Spain) approved the study protocol, project identification code C-4232-00, date 01/12/2018. ARRIVE guidelines [40], the guidelines outlined in EU directive 2010/63/EU and the Spanish regulations for animal experiments (Royal Decree 223/1988) were followed to perform the experiments.

### 2.10. Statistical Analysis

One-way ANOVA and Bonferroni post hoc test was used to analyse the changes in SBP and DBP obtained after the administration of the test peptides or the positive and negative controls. SPSS data analysis software was used (SPSS, Chicago, IL, USA).

## 3. Results

### 3.1. Preparation of a Water-Soluble Peptide Extract from Olive Oil

Unfiltered VOO was produced and an acetone/hexane extract was obtained from the oil. The yields calculated were 172.5 ± 83.6 mg of dried extract and 7.24 ± 4.1 mg of proteins per kg of olive oil (the content of proteins of the dried extract was approximately 4%). A peptide fraction was obtained from this extract containing 0.09 ± 0.02 mg of water-soluble peptides per kg of olive oil. These amounts represent only 1.2% of the peptides originally extracted with organic solvents.

### 3.2. Analysis and Identification of Olive Oil Peptides

**Figure 2:**
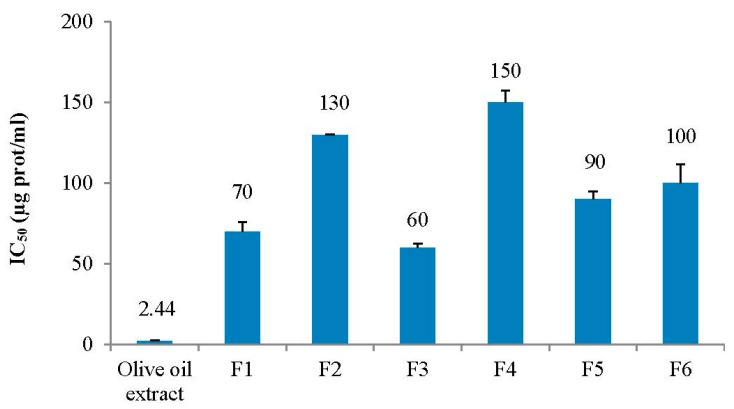
Angiotensin-converting enzyme (ACE) inhibitory activity (expressed as IC_50_) of water-soluble unfiltered virgin olive oil extract and of the fast protein liquid chromatography (FPLC)-purified fractions F1–F6. Data are expressed as mean values ± SD (bars).

The separation of peptides extracted from unfiltered VOO by gel filtration chromatography (FPLC) revealed several peaks at 280 nm (Figure 1). The ACE inhibitory activity of the water-soluble peptide extract injected in the FPLC and of the F1–F6 collected fractions was tested in vitro (Figure 2). The extract showed the highest activity (lowest IC_50_ value). Three major groups of fractions containing the main peptide peaks were selected, with molecular masses ranging from 5300–1600 Da (F3), 1600–700 Da (F4) and 700–280 Da (F5). The peptide fractions F3–F5 were analysed by nanoLC-Orbitrap-MS/MS and de novo sequencing with PEAKS studio software. Antihypertensive peptides usually have molecular masses between 3 kDa and 0.35 kDa so we focused of fraction F4. A total number of 149 de novo sequenced peptides were obtained with ALC confidence factor higher than 60% (not shown).

### 3.3. Peptide Selection and ACE Inhibitory Activity Determination

**Figure 3:**
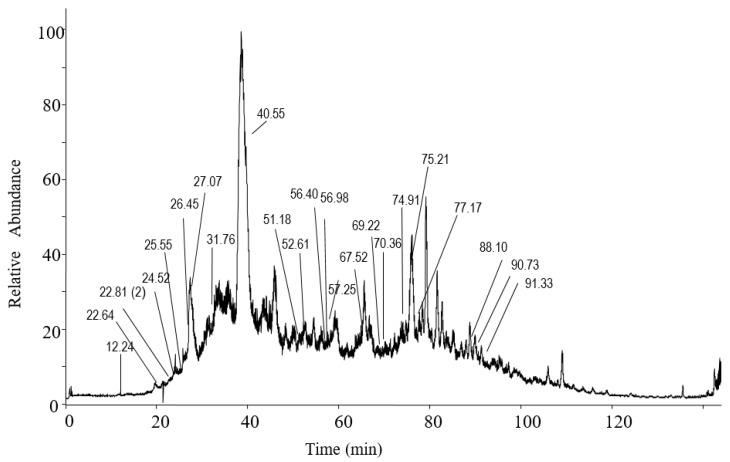
Analysis by nano- LC-Orbitrap MS/MS of water-soluble peptides extracted from unfiltered virgin olive oil. The total ion chromatogram and retention times of the selected ACE inhibitory peptides are shown. Brackets show the number of peptides with the same retention time.

**Figure 4:**
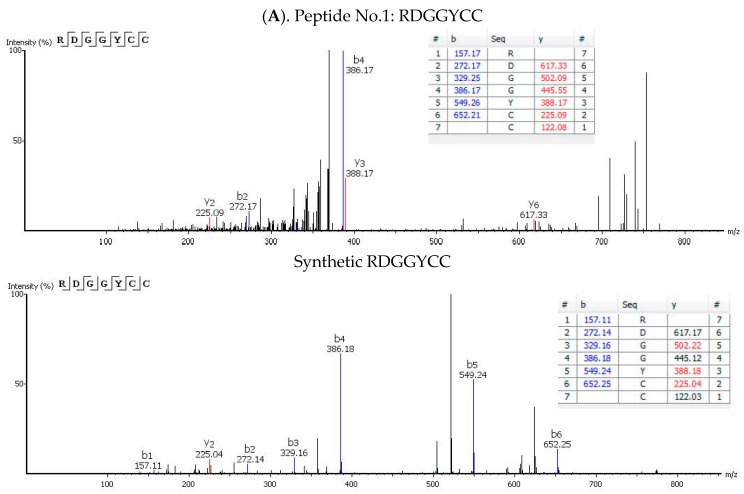

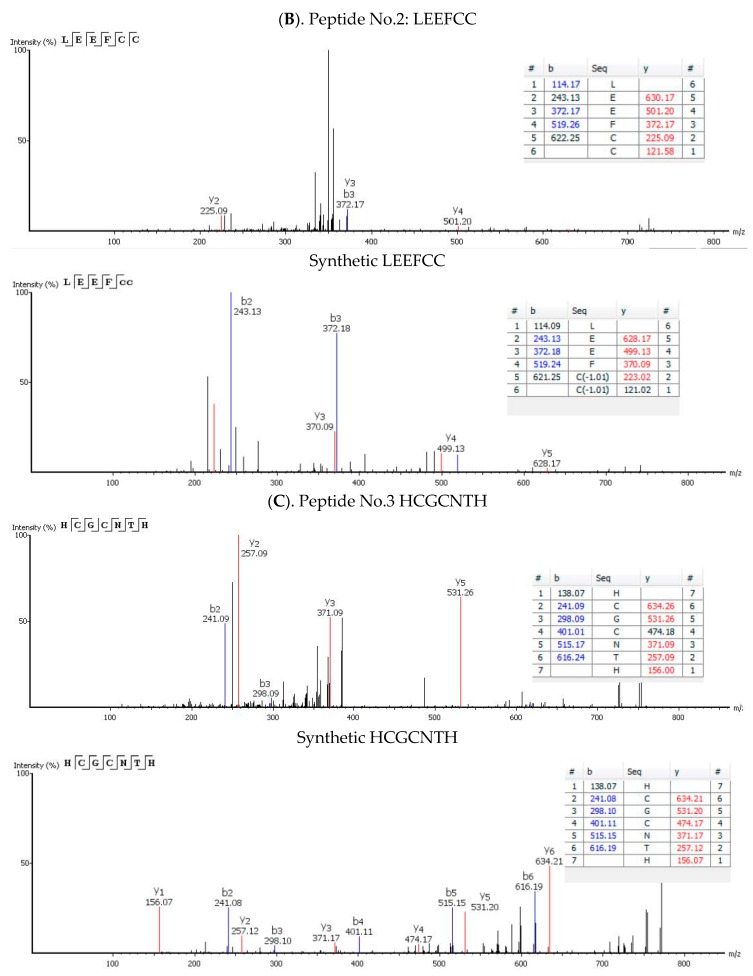

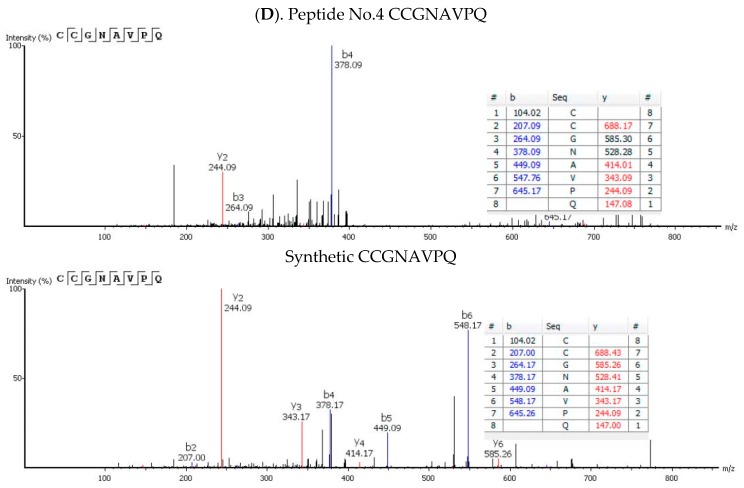
Fragmentation spectra of water-soluble peptides extracted from unfiltered virgin olive oil and of the corresponding purified synthetic peptides (below) analysed by nano- LC-Orbitrap MS/MS and PEAKS Studio software. (**A**), peptide No. 1, RDGGYCC; (**B**), peptide No. 2, LEEFCC; (**C**), peptide No. 3 HCGCNTH; and (**D**), peptide No. 4 CCGNAVPQ. Fragment ion tables for b and y ions (monoisotopic masses) are included.

Peptides with potential ACE inhibitory activity were selected as described in the methods section. Seventy-five peptides were independently detected in at least three different sample injections with an ALC value above 70%. Out of those, 23 peptides with potential ACE inhibitory activities were selected (Table 1). The peptides contained between 6 and 9 amino acids and molecular masses ranging 698–1017 Da. Their retention times are shown in Table 1 and Figure 3. The 23 sequences have not been reported before in any of the existing protein of peptide databases so, to the best of our knowledge, the peptide sequences are new. The sequences contained between 1 and 5 motifs with ACE inhibitory activity, with the exception of numbers 3, 7 and 10, which were selected because of their C-terminal hydrophobicity or amino acid charge. Peptide Nos. 5 and 6 have exactly the same sequence but with a different order of the amino acids L and P at the C-terminal end. They were both included in the selection because our analysis could not differentiate between the sequences at this level. The 23 peptides were chemically synthesised and the ACE inhibitory activity was quantified in vitro (Table 1). Peptide No. 1–3 and 5–8 possessed very high activity with IC_50_ values below 10 µm, whilst sequences Nos. 12–23 had IC_50_ values above 100. The activity of peptide No.17 was not determined because it was not water soluble. The highest IC_50_ (lowest activity) was detected for peptide No.18–23, with values above 350 µm. According to PEAKS software, the ALC value shows the estimated percentage of correct amino acids in the peptide sequence. An ALC value of above 90% is considered a great score. The four peptides with the highest ACE inhibitory activity also possessing an ALC value above 90% were selected for in vivo testing in SHR: peptide No.1, RDGGYCC; peptide No.2, LEEFCC; peptide No.3, HCGCNTH; peptide No.4, CCGNAVPQ. Synthetic purified peptides containing the same amino acid sequence as peptides No. 1–4 were obtained and analysed by nanoLC-Orbitrap MS/MS and processed with PEAKS studio software as well. Comparison of fragment ion tables and fragmentations spectra of olive oil peptides and synthetic peptides suggest that the sequences were confidently identified (Figure 4).

### 3.4. Antihypertensive Activity of Synthetic Virgin Olive Oil Peptides

**Figure 5:**
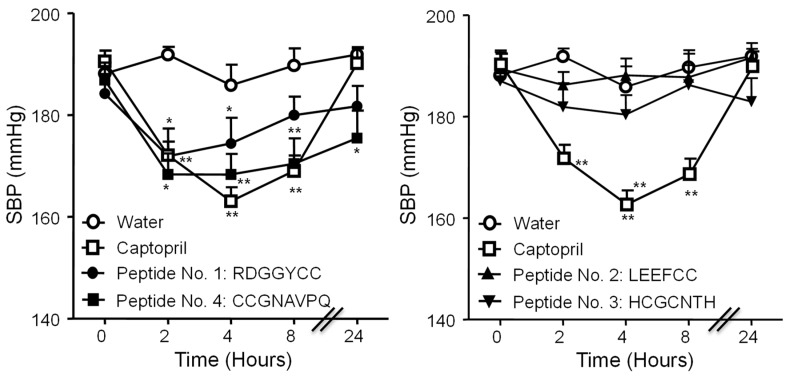
Systolic blood pressure (SBP) changes (mmHg) detected in spontaneously hypertensive rats (SHR) at baseline and 2, 4, 8 and 24 h after the administration of a 25 mg/kg BW dose of synthetic peptides No. 1 (RDGGYCC, ●), No. 2 (LEEFCC, ▲), No.3 (HCGCNTH, ▼), No.4 (CCGNAVPQ, ■), Captopril (50 mg/kg of BW, □), or water control (○). * *p* < 0.05; ** *p* < 0.01 vs. control.

We tested the antihypertensive effects of the synthetic peptides No. 1–4. We selected those sequences because of their high activity in vitro and the number of potentially active ACE inhibitory activity fragments. A dose of 25 mg/kg of BW was administered to the animals. The average initial values of SBP (197.8 ± 3.9 mmHg *n* = 30) showed that the animals suffered from high blood pressure at the beginning of the study. Peptides No. 1 and No. 4 produced SBP maximum reductions of 10 and 12% (19 and 23 mmHg), respectively, 2 h after their administration, compared with controls, similar to the reduction produced by Captopril (Figure 5). The effect of peptide No. 1 began to revert at 4 h and disappeared at 8 h, whilst the antihypertensive effect of peptide No. 4 was sustained at times 4, 8 h and was even significant at 24 h, when the Captopril group had returned to basal levels. So, at the doses administered, the effect of peptide 4 was more prolonged than that of Captopril. Only non-significant reducing trends were detected for peptide No. 2 and No. 3 compared with controls.

## 4. Discussion

In this study we have shown that unfiltered VOO contains small peptides with specific ACE inhibitory activity and antihypertensive effects on the SHR animal model of hypertension. We also characterised some sequences of ACE inhibitory and antihypertensive peptides from this oil. As we described previously [28], avoiding the filtration step of olive oil production was an important factor for the isolation of the peptides (extensively used by industry to eliminate oil turbidity). For this reason, we produced our own unfiltered VOO to be able to isolate their peptides content for this research. For the initial extraction of peptides from VOO we tested many different solvent mixtures and conditions. Organic solvents produced the best results, particularly the extraction with acetone: hexane, which has been successfully used in the past for the extraction of proteins in refined and non-refined oils [26]. This is probably due to the nature of the food matrix. After the extraction step, the organic solvents were eliminated and the dried residue was extracted with water only. Only a small fraction (water-soluble, about 1%) of the proteins/peptides present in the olive oil of the study were extracted, so there might be other peptides present in olive oil with biological activity, yet to be studied. We only focused on the water-soluble fraction because we had the intention of investigating in vivo the effects of pure peptide sequences present in unfiltered VOO. The use of organic solvents is incompatible with the SHR model of hypertension, so one limitation of this study was indeed that we studied only the water-soluble peptide fraction of the oil.

Different methodologies have been used to determine the concentration of proteins in olive oils but the results reported are controversial. Some authors reported values of 0.05–2.4 mg/kg [24] or 0.1–0.5 mg/kg [25] but other studies showed much higher values of 11–43 mg/kg of oil [23]. One of the first studies investigating the protein content of olive oils reported that the proteins were almost exclusively composed of a 4.6 kDa polypeptide with oleosin-like characteristics [24]. A later study showed the presence of olive oil proteins with molecular weight above 30 kDa, without finding any evidence of the 4.6 kDa protein [26]. In a recent article [41], the authors showed that the 4.6 kDa protein was an artefact from the filter paper used to clarify the extracts and they only found proteins of molecular weights higher than 10 kDa. Using a protein extraction method with acetone:hexane, which showed the best extraction results and the lowest interference problems, the concentration of proteins in virgin olive oil was reported to range 0.2–0.6 mg/kg [26,27]. This is at least ten times less than the protein concentration obtained by us, measured in unfiltered olive oil. An explanation of the low value obtained by those authors could be that they used commercial (filtered) olive oils for the extraction.

The low molecular weight protein fraction of olive oil has been poorly studied so far. In our study, the olive oil extracted proteins/peptides were subjected to separation by molecular filtration using a column specifically designed for peptide separation. We only focused on the nano LC-Orbitrap-MS/MS analysis of fraction F4 (700–1600 Da) because antihypertensive peptides (obtained by hydrolysis) typically have molecular weights between 350 Da and 3000 Da [42]. The F6 fraction (280–150 Da) showed some ACE inhibitory activity possibly because of the presence of some dipeptide species possessing this activity. Besides, the contribution of low molecular mass water-soluble compounds possessing activity cannot be ruled out. Another limitation of the present study is the difficulty to identify olive oil peptides and proteins. No database for *Olea europaea* is currently available so the only solution for the determination of the peptide sequences present in the samples was de novo sequencing. This consists of a prediction of amino acid sequence using the MS/MS data and fragmentation spectra obtained from the sample, by comparison with a database of previously analysed peptides. The predicted sequence is rated by the average local confidence parameter (shown as %), which shows the percentage of correct amino acids of the sequence (according to Peaks software, the prediction is great if over 90%). We used synthetic purified peptides as standards to compare the fragment ions and fragmentation spectra with the identified peptides. The analysis of synthetic peptides using the same methodology provided almost identical fragment ion tables for b and y ions to the ones detected in the olive oil samples and also similar fragmentation spectra, suggesting that sequences Nos. 1–4 were bona fide identified peptides.

The study of ACE inhibitor peptides in foods is a very active area of research and there are a few available data bases with information on peptide sequences and biological activity. We used the BIOPEP data base to assist with our selection of peptides because it uses previous published research providing information about the number of ACE inhibitor motifs of a peptide sequence and calculates the peptide’s theoretical activity. Apart from the ACE inhibitory activity, the data base detected additional potential activities of our selected peptides, including antioxidant (Nos. 9 and 11), anti-thrombotic and anti-amnestic (Nos. 19, 20 and 22), immuno-modulating (Nos. 5 and 6), hypoglycaemic (Nos. 1, 3–23) and activators of ubiquitin-mediated proteolysis (Nos. 8, 14, 16, and 20), which could be the subject of other studies, but is out of our scope. Most of our selected peptides possessed measurable ACE inhibitory activity in vitro. However, the ACE inhibitory activity of the four synthetic peptides tested in vitro did not correlate with the activity tested in SHR. Indeed, there appears to be no relationship between the extent of SBP decrease and the IC_50_ values [43]. This is because the capacity of peptides to produce antihypertensive effects mainly depends on their ability to reach blood and organs and this is conditioned by their bioavailability. Proteins and peptides are metabolised to single amino acids but some peptides with two, three or more amino acid residue lengths can be absorbed directly from the digestive tract into the blood. For example, the tri-peptides IPP and VPP are absorbed intact into the circulation and reduce SBP in SHR and in humans [44,45].

The process of development of high blood pressure in humans presents analogies with the development of hypertension in SHR. For this reason, the SHR model has been extensively used in the evaluation of food peptides with antihypertensive potential [21]. Two of the four synthetic peptides tested in SHR showed antihypertensive activity at the doses administered. The ACE inhibitory activity of peptide SEQ No. 1 (RDGGYCC) was the highest of the selected peptides and also possessed an antihypertensive effect. It contains four dipeptides (DG, GG, GY, DG) with previously reported activity [46], however, the antihypertensive effect of the terminal tripeptide YCC has not been described before. SEQ No. 4 (CCGNAVPQ) did not show a very high ACE inhibitory activity but the SBP reducing effect was remarkable, similar to that of Captopril. Likewise, the bovine casein-derive peptide KVLPVPQ, containing the same C-terminal tripeptide, also showed low in vitro ACE inhibitory activity but possessed high SBP reducing effect in SHR [47]. This observation suggests that an in vivo process of proteolysis of this peptide rendering VPQ could be important for its antihypertensive activity. However, two important limitations of this research were the absence of bioavailability studies with the synthetic peptides used in the animal model and the lack of ACE-inhibitory activity measurements in vivo. Those studies will be the focus of future research. Elevated blood pressure is one of the few well-documented primary risk factors of cardiovascular disease [48]. Relatively small reductions in SBP (10–12 mmHg) have been reported to substantially reduce the cardiovascular risk in humans [49]. Regarding the magnitude of the effect, the SBP reductions of 10–20 mmHg produced by SEQ No.1 and No.4 could be relevant and deserve further investigation in volunteer subjects. The SBP decrease obtained with the pure synthetic peptides is very similar to that obtained in other studies administering comparable amounts of pure peptides obtained from dairy proteins [50,51,52]. A number of those peptide compositions from dairy foods with demonstrated antihypertensive activity in humans constitute the basis of commercially available functional foods. Perhaps the consumption of unfiltered VOO containing peptides could produce benefits regarding the control of blood pressure in humans. We are in the process of investigating the effects of unfiltered VOO, naturally containing the above peptides, in humans suffering from pre-hypertension.

We studied the most frequent Spanish olive oil variety (Picual), but only one variety, which is another limitation of this research. Other varieties may show different results. Apart from VOO, a likely source of olive peptides could be olive oil waste [32], which produces environmental problems. In view of our results, the study of the peptide composition of other varieties of VOO and of olive oil by-products should be investigated further.

## 5. Conclusions

In conclusion, unfiltered VOO naturally contains low molecular weight peptide sequences with specific ACE inhibitory and antihypertensive activity with potential nutritional and pharmaceutical applications.

The work reported in this manuscript has the international patent reference numbers EP153800492015 and PCT/EP16/078162 [53].

## Figures and Tables

**Table 1:** Water-soluble peptides with ACE inhibitory activity identified in the olive oil extract using Peaks studio software and de novo sequencing. N, number of amino acids; RT, retention time; ALC, average local confidence; ACEi activity, angiotensin converting enzyme inhibitory activity; IC_50_ (µm), peptide concentration necessary to inhibit 50% of ACE activity, expressed in µm; A, number of sequences with ACEi activity/N; B, potential biological activity of the peptide (µm^−1^) theoretically calculated from previous ACEi referenced data. A and B values obtained from the BIOPEP data base (http://www.uwm.edu.pl/biochemia/index.php/pl/biopep).

Sequence Number	Peptide Sequence	N	RT (min)	Mass	ALC (%)	ACEi Activity IC_50_ (µm) ± SD	N° Seq with ACEi Activity	A	B
SEQ No.1	RDGGYCC	7	69.22	772.26	92	0.84 ± 0.02	4	0.57	0.1222
SEQ No.2	LEEFCC	6	31.76	742.27	94	1.85 ± 0.16	1	0.17	0.0074
SEQ No.3	HCGCNTH	7	26.45	770.26	97	2.58 ± 0.26	0	0	ND
SEQ No.4	CCGNAVPQ	8	25.55	790.31	95	39.56 ± 2.09	4	0.50	0.0008
SEQ No.5	YGCGCDLP	8	22.81	826.30	75	3.51 ± 0.21	2	0.25	0.0261
SEQ No.6	YGCGCDPL	8	22.81	826.30	75	1.64 ± 0.02	2	0.25	0.0005
SEQ No.7	YMHCYF	6	57.25	862.31	74	3.20 ± 0.23	0	0.00	ND
SEQ No.8	WAAGYCC	7	77.17	772.27	77	6.88 ± 0.30	3	0.43	0.0010
SEQ No.9	EECHAH	6	90.73	724.26	83	11.80 ± 1.99	1	0.17	ND
SEQ No.10	YESMADPCS	9	12.24	1017.34	71	24.12 ± 0.03	0	0	ND
SEQ No.11	DMCPHD	6	52.61	732.22	78	38.28 ± 0.41	1	0.17	ND
SEQ No.12	LEFTSSY	7	27.07	845.38	93	107.42 ± 14.69	2	0.29	0.0024
SEQ No.13	RQTCDM	6	70.36	768.29	92	108.80 ± 20.46	0	0	ND
SEQ No.14	LALAPTN	7	67.52	698.40	72	135.29 ± 13.52	5	0.71	0.0424
SEQ No.15	RPVPPVF	7	91.33	810.48	70	229.55 ± 36.46	5	0.71	0.0325
SEQ No.16	LALSAPVP	8	88.10	766.46	71	305.04 ± 10.88	3	0.38	0.0012
SEQ No.17	LQFCCY	6	75.21	775.30	83	ND	1	0.17	ND
SEQ No.18	KRRPPTT	7	56.98	854.51	84	>390	6	0.86	0.0041
SEQ No.19	EAPGGYDD	8	40.55	822.30	70	>405	6	0.75	0.0973
SEQ No.20	LAPGGDMD	8	56.40	790.32	71	>422	6	0.75	0.0367
SEQ No.21	YEAYPS	6	22.64	728.30	73	>460	4	0.67	0.0090
SEQ No.22	EDHGPM	6	24.52	700.25	78	>476	3	0.50	0.0105
SEQ No.23	MSMAPY	6	51.18	698.28	80	>480	1	0.17	0.0007

## Abbreviations

ACE	angiotensin converting enzyme
BW	body weight
DBP	diastolic blood pressure
SDP	systolic blood pressure
SHR	spontaneously hypertensive rats
